# Descriptive Epidemiology of Female Suicides by Race and Ethnicity

**DOI:** 10.1007/s10900-024-01368-z

**Published:** 2024-06-09

**Authors:** James H. Price, Erica Payton Foh

**Affiliations:** 1 University of Toledo, Toledo, OH 43606 USA; 2 University of North Carolina at Greensboro, Greensboro, NC USA

**Keywords:** Suicide, Females, Suffocation, Firearms, Race, Ethnicity

## Abstract

Each year millions of females develop serious mental illnesses (SMI), which are major risk factors for suicides. Using the Web-Based Injury Statistics Query and Reporting System (WISQARS) for the years 2000, 2010 and 2020, we found in 2020 9,428 females (almost 190/week) committed suicide, losing 328,653 years off potential life before age 80 years. There were pronounced increases in female suicides from 2000 to 2020 across all racial and ethnic groups. The greatest number of suicides were in non-Hispanic white females, but the highest rate of suicides was in non-Hispanic American Indians /Alaska Natives, and in females 15–24 years of age. The West had the highest female suicide rates, with methods used to commit suicides varying by census regions and race and ethnicity. Suffocation to commit suicide increased for most racial and ethnic groups and poisonings decreased for most groups between 2000 and 2020, These underscore the need for targeted primary prevention of suicides for females based on age, geographic location and method of suicide, to mitigate female suicides improved access (e.g. geographically and financially) to mental health care services is essential.

## Introduction

Suicide is one of the leading causes of death in the United States (U.S.), an ever increasing public health crisis with no end in sight [1, 2]. The increases in suicides have occurred in both males and females, in all age groups, and in various racial and ethnic groups [2–7]. From an historical perspective, we find females have consistently had greater rates of suicide ideation, suicide planning, and suicide attempts than males [8, 9]. However, males in the U.S. have a greater death rate by suicides than females [9], a rate almost four times that of females [2]. In Europe, there are similarities of suicidal behaviors with the U.S.; females engage in greater rates of suicide ideation and attempts and males die of suicide more often [10, 11]. In the U.S. in 2020, over 1.2 million individuals over the age of 18 reported they had attempted suicide and 44, 290 died by suicide for a rate of 17.26 per 100,000 [12, 13]. According to this data, suicide attempts occur at a rate approximately 27 times the rate of suicide deaths.

Since 1999, there have been major increases in suicides and the increase in female suicides has been greater than the increase in male suicides [2, 14]. Females seem to be at greater risk for suicidal behaviors because of psychopathologies (e.g. depressive disorders, anxiety disorders, eating disorders, PTSD and selected psychosocial stressors (e.g. domestic violence, physical/emotional/sexual abuse, sexual prejudice, incarceration, financial instability, etc.) [15, 16]. The suicide paradox of females attempting suicide more often than males and dying from their attempts less often than males needs further explanation.

A study of suicides in Europe found the higher lethality of suicide attempts in males was best explained by males choosing more lethal suicide methods 69% of the time and females did so 42% of the time [11]. In addition, they found higher lethality of suicidal acts for males even when females used the same methods. This study also assessed the intentionality of suicide attempts using a rating based on the Feuerstein scale. The intention to die by their suicides and suicide attempts was greater in males than females but did not reach statistical significance (*p* = .09). However, another group of researchers analyzed data from the same database and concluded that “serious suicide attempts” occurred significantly (*p* < .001) more often in males than in females [10].

Research on suicide in the U.S. has focused on changing rates of suicides over time, on constructs between male and female suicides, and the different methods used to commit suicide (especially firearms). Very limited suicide research has specifically focused on females as the central subject [17]. To research a better understanding of suicide in females in the U.S. we need more information on suicides by females. More specifically, how different methods of suicide vary by race/ethnicity, variations in suicide rates for the various race/ethnicities by geographic locations, and suicides by race/ethnicity and age. Recently, research examined many of these issues in Black females [18, 19]. The current study examines the aforementioned questions by examining suicides for four racial groups and Hispanic ethnicity in females. To our knowledge this will be the first study to explore these issues contrasting the findings in various demographic groups of females.

## Methods

The Centers for Disease Control and Prevention’s (CDC) Web-based Injury Statistics Query and Reporting System (WISQARS) with repeated cross-sectional mortality data for females ages 1 to 85^+^ years was the database for this study. The years 2000, 2010, and 2020 were used to explore the changing characteristics of female suicides. WISQARS is a national, regional and state based mortality database that is a publicly available interactive deidentified database. Because the database consists of deceased individuals with all their data deidentified this study was exempted from Institutional Review Board assessment.

We used descriptive statistics (frequencies, percentages, and age-adjusted rates per 100,000 females) to describe the changing characteristics of females by year (2000, 2010, 2020), age (1–14, 15–24, 25–44, 45–64, 65–85^+^ years), race (non-Hispanic Asian/Pacific Islander, non-Hispanic American Indian/Alaska Native, non-Hispanic Black, non-Hispanic white), ethnicity (non-Hispanic (NH) vs. Hispanic), geographic location (Northeast, South, Midwest, West), and methods of suicide (firearms, suffocation, poisoning, other). Geographic locations were based on the CDC’s census regions rather than individual states because of inadequate numbers (20 or less) of suicides for some races and ethnicities spread across 50 states. The CDC has aggregated the states into four census regions: Northeast (CT, MA, ME, NH, NJ, NY, PA, RI, VT), South (AL, AR, DC, DE, FL, GA, KY, LA, MD, MS, NC, OK, SC, TN, TX, VA, WV), Midwest (IA, IL, IN, KS, MI, MN, MO, ND, NE, OH, SD, WI)., and West (AK, AZ, CA, CO, HA, ID, MT, NM, NV, OR, UT, WA, WY).

Key demographic characteristics of deceased females in the database were determined by the CDC. Additionally, years of potential life lost before age 80 years (YPLL 80) were summed across all the ages of deceased females by suicide to obtain the total numbers of years prematurely lost for the years 2000, 2010 and 2020 [20, 21]. The decision to use the age of 80 years for all racial and ethnic groups was based on the assumption that virtually every U.S. citizen should be able to reach that age by engaging in healthy behaviors, having adequate social and financial resources, and with adequate access to healthcare services. In other words, while life spans by race and ethnicity currently varies in the U.S. we reject the current life spans as the basis for determining the number of years of potential life females of various races and ethnicities lose to suicide, in large part, due to the disparities in the social determinants of life.

## Results

In the decade from 2000 to 2010 there was 41% more female suicides compared to 2000, in 2020 there was a 16.6% increase from 2010 in female suicides (Table 1). Because the largest population of females in the United States is NH whites they consistently had the largest number of suicides. However, when the various races and ethnicities are examined we find NH AI/AN have consistently had the highest age adjusted suicide rate per 100,000 individuals (Table 1). An exploration of female suicides by age found by 2020 the highest female suicide rates were in the 15–24 year olds for all racial groups, except NH white females where it was ages 45–64 across all three years (Table 2). Female suicide rates increased from 2000 to 2020 for all age groups and races and ethnicities, except for the age group 65–85^+^ for NH Blacks and Hispanics where the rates declined. In 2020, the absolute highest suicide rate by age groups and race and ethnicities was for 15–24 year old NH AI/ANs (20.11 per 100,000), a rate 3 to 4 times greater than other racial and ethnic groups.

**Table 1:** Rates and percents of female suicide by race and ethnicity

Date				
Race/ethnicity	N	Age adjusted rate	Percent of suicide	Percent of female population
2020				
NH Asian/PI	439	3.81	4.7	6.6
NH AI/AN	151	10.78	1.6	0.8
NH Black	661	2.92	7.0	13.6
Hispanic	870	2.83	9.2	18.2
NH White	7,283	6.92	77.2	60.8
Totals	9,428	5.5	100	100
2010				
NH Asian/PI	299	3.43	3.7	5.4
NH AI/AN	118	9.03	1.5	0.8
NH Black	379	1.85	4.7	13.1
Hispanic	493	2.11	6.1	15.8
NH White	6,772	6.25	83.7	64.8
Totals	8,087	5.0	100	100
2000				
NH Asian/PI	160	2.80	2.8	4.1
NH AI/AN	58	4.66	1.0	0.8
NH Black	322	1.79	5.6	12.9
Hispanic	262	1.71	4.6	12.0
NH White	4,905	4.66	85.6	70.2
Totals	5,732	4.0	100	100

NH = non-Hispanic; PI = Pacific Islander; AI = American Indian; AN = Alaska Native

Source: CDC WISQARS

**Table 2:** Female suicides by age and race/ethnicity

Race/ethnicity			Years			
	2020		2010		2000	
Age (yrs)	n	rate	n	rate	n	rate
Non-Hispanic Asian/PI						
1–14	7*	0.40	2*	0.14	3*	0.27
15–24	94	7.12	39	3.42	25	2.85
25–44	134	3.78	121	4.29	67	3.20
45–64	130	4.69	101	4.86	40	3.18
65–85^+^	74	4.71	36	4.32	25	5.01
Non-Hispanic AI/AN						
1–14	8*	2.96	5*	1.72	1*	0.34
15–24	41	20.11	36	16.74	16*	8.16
25–44	77	19.88	41	11.79	33	9.21
45–64	22	6.49	28	8.64	8*	3.47
65–85^+^	0*	0.00	8*	7.21	0*	0.00
Non-Hispanic Black						
1–14	35	0.82	20*	0.47	5*	0.12
15–24	159	5.06	69	2.08	64	2.25
25–44	292	4.58	164	2.91	153	2.69
45–64	140	2.55	109	2.16	77	2.17
65–85^+^	34	1.09	17*	0.82	23	1.31
Hispanics						
1–14	56	0.78	22	0.33	7*	0.15
15–24	225	4.59	131	3.11	60	1.98
25–44	380	4.30	182	2.40	115	2.07
45–64	167	2.66	123	2.79	63	2.49
65–85^+^	42	1.53	35	2.19	17*	1.67
Non-Hispanic whites						
1–14	105	0.74	38	0.25	46	0.27
15–24	682	6.06	552	4.44	401	3.28
25–44	2,273	9.25	2,114	8.56	1,922	6.68
45–64	2,889	10.52	3,210	10.74	1,775	7.25
65–85^+^	1,334	5.75	857	4.69	7.61	4.41

*numbers based on 20 or fewer individuals may be unreliable

Source: CDC WISQARS

The distribution of female suicides across the four census regions of the U.S found the Northeast had the lowest suicide rates across all races and ethnicities (Table 3). By 2020 the highest rates of female suicides for all the groups, except NH AI/ANs, was the West. Surprisingly, the highest suicide rate in 2020 was in the Midwest. Furthermore, the only census regions in 2020 with double digit female suicides was for NH AI/ANs in the Midwest and West. The various methods used to commit suicide was examined for the various races and ethnicities (Table 4). Across all three years, NH Blacks and NH whites were the groups most likely to commit suicide with firearms. In contrast, NH AI/PIs, NH AI/ANs, and Hispanics were most likely to use suffocation. The group that was most likely to use poisonings was NH whites.

**Table 3:** Female suicides rates by census regions and race/ethnicity

		Years		
Race/ethnicity	Census region	2020	2010	2000
		Age adjusted rates		
Non-Hispanic Asian/PI	NE	2.85	3.18	2.00
	S	3.94	3.11	1.98
	MW	3.86	5.06	3.71
	W	4.13	3.38	3.10
Non-Hispanic AI/AN	NE	1.52*	1.23*	0.00*
	S	6.88	6.40	3.34*
	MW	14.70	12.37	5.16*
	W	13.30	10.56	5.97
Non-Hispanic Black	NE	2.15	1.57	1.93
	S	2.48	1.53	1.55
	MW	3.86	2.27	1.93
	W	5.26	3.67	2.73
Hispanic	NE	1.69	1.78	1.75
	S	2.90	2.07	1.63
	MW	3.05	2.46	1.49*
	W	3.10	2.20	1.76
Non-Hispanic Whites	NE	5.11	4.41	3.03
	S	7.70	6.98	5.72
	MW	6.08	5.34	3.76
	W	8.30	8.03	5.78

*age adjusted rates based on 20 or fewer individuals may be unreliable

Source: CDC WISQARS

**Table 4:** Racial/ethnic variations in suicide methods used by females

Race/ethnicity	Firearms		Suffocation			Poisoning		Other**		
Year	n	(%)	n	(%)		n	(%)	n	(%)	
2020										
Non-Hispanic Asian/PI	48	(10.9)	204		(46.5)	103	(23.5)	84		(19.1)
Non-Hispanic AI/AN	31	(20.5)	82		(54.3)	31	(20.5)	7*		(4.6)
Non-Hispanic Black	206	(31.2)	197		(29.8)	159	(24.1)	99		(15.0)
Hispanic	204	(23.4)	398		(45.7)	170	(19.5)	98		(11.3)
Non-Hispanic White	2,619	(36.0)	1,852		(25.4)	2,225	(30.6)	587		(8.1)
2010										
Non-Hispanic Asian/PI	22	(7.4)	172		(57.5)	58	(19.4)	47		(15.7)
Non-Hispanic AI/AN	0*	(0)	44		(37.3)	39	(33.1)	35		(29.7)
Non-Hispanic Black	111	(29.3)	110		(29.0)	111	(29.3)	47		(12.4)
Hispanic	94	(19.1)	202		(41.0)	125	(25.4)	72		(14.6)
Non-Hispanic White	2,166	(32.0)	1,369		(20.2)	2,680	(39.6)	557		(8.2)
2000										
Non-Hispanic Asian/PI	21	(13.1)	80		(50.0)	28	(17.5)	31		(19.4)
Non-Hispanic AI/AN	23	(39.7)	16*		(27.6)	15*	(25.9)	4*		(6.9)
Non-Hispanic Black	124	(38.5)	60		(18.6)	86	(26.7)	52		(16.1)
Hispanic	76	(29.0)	84		(32.1)	66	(25.2)	36		(13.7)
Non-Hispanic White	1,885	(38.4)	710		(14.5)	1,859	(37.9)	451		(9.2)

**Other = immolation, drowning, jumping, moving vehicles, cutting, etc

*Age adjusted rates based on 20 or fewer individuals may be unreliable

Source: CDC WISQARS

From 2000 to 2010 the use of firearms as a percent of all suicides decreased for all racial and ethnic groups. In contrast, the percent of all suicides committed by suffocation increased, except for NH AI/PIs and the percent of suicides by poisonings decreased for all groups except NH AI/PI (Table 4). An examination of methods of suicide by various regions found that firearms are most likely to be used in the South and least likely in the Northeast (Table 5). Suffocation has consistently been used most often in the Northeast and least often in the South. Poisoning to commit suicide decreased as a percent of suicides from 2000 to 2020. An assessment of years of potential life lost before age 80 due to female suicides found pronounced increases over the three years. In 2000 there were 198,287 years of life lost and in 2010 it increased to 274,332, a 38.4% increase. From 2010 to 2020, the number of female years of life lost to suicide (328,653) increased by 19.8%.

**Table 5:** Regional variations in suicide methods used by females

	Census	Firearms			Suffocation		Poisoning		Other	
	region	n	(%)		n	(%)	n	(%)	n	(%)
2020	NE	220		(18.0)	392	(32.2)	438	(35.9)	169	(13.9)
	S	1567		(42.2)	937	(25.2)	947	(25.5)	264	(7.1)
	MW	590		(30.5)	602	(31.1)	577	(29.8)	168	(8.7)
	W	735		(29.5)	811	(31.7)	732	(28.6)	27.9	(10.9)
2010	NE	172		(15.6)	348	(31.5)	430	(38.9)	155	(14.0)
	S	1,248		(40.6)	542	(17.6)	1,043	(33.9)	244	(7.9)
	MW	418		(24.7)	462	(27.3)	681	(40.2)	134	(7.9)
	W	592		(26.8)	549	(24.8)	872	(39.5)	197	(8.9)
2000	NE	156		(19.9)	179	(22.8)	303	(38.6)	147	(18.7)
	S	1,080		(46.6)	247	(10.7)	818	(35.3)	173	(7.5)
	MW	379		(32.6)	227	(19.6)	433	(37.3)	122	(10.5)
	W	517		(35.2)	302	(20.6)	513	(34.9)	136	(9.3)

*Other = immolation, drowning, jumping, moving vehicles, cutting, etc

Source: CDC WISQARS

## Discussion

Findings from our study show that NH white females constitute the largest number of female suicides but NH AI/AN females had the highest age adjusted suicide rate in 2000, 2010, and 2020. Furthermore, the suicide rate for females increased for all five racial and ethnic groups and over the three time periods. These increases may be due to an increasing lack of resources to help reduce suicide rates, an increase in number or intensity of risk factors for suicide, and/or suicide interventions that do not work adequately. The age group in 2020 with the highest age adjusted rate of suicides was those of 15–24 years for four of the five racial and ethnic groups studied, but not for NH white females (45–64 years). This difference in age for NH white females may be due to increased access to mental health care in the younger ages or possibly different risk factors for suicide impacting the older age group.

Age adjusted female suicide rates in 2020 were highest for the various races and ethnicities by census regions as follows: West (NH A/PI, NH Black, NH white and Hispanics) and the Midwest (NH AI/AN). We also found a pronounced increase from 2000 to 2020 in use of suffocation for female suicides. We hypothesize that the ubiquitous nature of items available to commit suffocation and the less gruesome nature of this method compared to firearms may be driving the pronounced increase. Furthermore, females who may be impulsive and confronting excessively stressful risk factors for suicide would not need intentional planning in using suffocation as their method of suicide.

It should be noted that the choice of method to commit suicide depends on a multitude of variables: accessibility, affordability, familiarity, biological factors (e.g. presences of illness), and social factors (“masculine” methods, level of trauma to survivors, cultural constraints) [11]. By 2020, results indicated that firearms were used as a method of suicide most often by NH whites and NH Blacks, suffocation was used most often by NH AI/ANs, and poisonings were used most often by NH white females. A large share of NH Blacks used a variety of “other” methods to commit suicide. By 2020 methods used to commit suicide varied by census regions, with the use of firearms and poisonings most common in the South and Northeast, respectively. In contrast, suffocation was nearly evenly spread across the Northeast, Midwest and West.

Previous studies on female suicides have explored portions of our topics on suicides. A study of suicides among Black females found their rates from 1999 to 2020 had a pronounced increase and that the highest rates of suicide were in the age groups 15–24 and 25–34 years, similar to our findings [18]. Another study of female suicides used 9 census divisions and found suffocation was the leading method of suicide in the northern divisions and we found the census region with the highest proportion of suffocation suicides to be in the Northeast [22]. The authors hypothesize that the increase in use of suffocation might be due to the increased information on how to die found on social media and the Internet. In addition, the study found the lowest age-adjusted female suicide rates in the Northeast and the highest was in the Mountain region followed by the West North Central region, results similar to our findings.

A multitude of variables (risk factors) have been correlated with females who commit suicide. The correlates with suicide have become so numerous that computer programs are being explored which can combine different combinations of risk factors in an attempt to better predict who will commit suicide. The variables that correlate across multiple studies with female suicides are usually mental disorders and psychosocial stressors [23]. Women report higher rates of major depression, borderline personality disorder, PTSD and eating disorders, all conditions associated with increased suicide risk [24–28]. The aforementioned mental disorders are part of a subset of mental disorders known as serious mental illnesses (SMIs) and are especially dangerous as risk factors for suicides, especially if left untreated. In 2020, 27% (9.1 million) of females with a mental illness (33.6 million) had an SMI and the prevalence varied by age (18–25 years 13%, 26–49 years 8.4%, and 50^+^ years 4.2%) [12]. During the previous year 30.1% of females with SMIs did not receive any treatment for their disorder.

Furthermore, lack of treatment for SMIs was higher in some racial and ethnic groups. SMIs can cause disruptions in individuals’ thinking and behaviors. These impairments will cause many of these individuals to expend all of their financial resources, spending down to poverty [28]. Poverty is itself one of many social determinants of mental health risk factors [29]. Addressing mental health disorders requires “upstream” interventions before mental health disorders emerge, and many of these disorders emerge in the teenage years. Universal public health education that includes mental health literacy is essential with a focus on understanding mental disorders, when to get help, and how these disorders are treated. Additionally, a focus on development of social skills (effective coping and problem-solving skills, decision-making, developing high self-esteem and a strong sense of cultural identity) can assist females in confronting stresses that are threats to their mental health. We believe by developing protective factors in adolescents and young adults these factors are more generalizable methods of confronting the multitude of mental health risk factors confronting females.

Even in the best circumstances there will be females that develop mental health disorders. These females will need physical and financial access to mental healthcare providers who are well trained on suicide-related interventions. Mental health care providers who perceive their training in working with suicidal patients as very good, feel more confident in assessing such patients and less discomfort in working with suicidal patients [30]. However, a realistic assessment of well-trained mental health professionals in suicide matters will not be a panacea for eliminating suicides. Mental health experts ability to predict if someone is going to attempt suicide is no better than chance [31]. The presence of risk factors for suicide in an individual may increase suicidal risk for hours, a few days, or a couple of weeks, not for months or years, making predictions likely to result in false positives and false negatives.

Finally, there are a number of potential limitations to this study that should be noted [32]. The WISQARS database did not contain numerous demographic variables (e.g. education levels, economic status, etc.) that could provide additional insights into suicides. Potential misclassification of suicides (e.g. whether a drug poisoning is a suicide or accidental overdose) could be partially responsible for racial and ethnic disparities in methods of suicide. Data in some tables are marked with asterisks, indicating inadequate numbers of suicide to rely on the numbers as valid representations of demographic differences. Additionally, incorrect identification of some suicide victims (e.g. AI/AN females and Hispanic females married to males of other racial or ethnic groups) could lead to underestimation of suicides in their correct racial or ethnic group.

## Conclusion

The findings of our study have public health and practical implications which may assist public agencies that address suicides. We found pronounced increases in female suicides across all racial and ethnic groups and the highest suicide rates were in those 15–24 years for all except NH whites (45–64 years). Furthermore, the West had among the highest female suicide rates, with methods used to commit suicides varying by census regions and race and ethnicity. Suffocations increased for most racial and ethnic groups and poisonings decreased for most groups. In 2020, females lost almost 329,000 years of life before the age of 80. Consideration of targeted primary prevention of suicides for females seems warranted. To mitigate female suicides improved access to mental health care services is essential.
